# Laparoscopic versus open liver resection: a meta-analysis of long-term outcome

**DOI:** 10.1111/hpb.12117

**Published:** 2013-05-15

**Authors:** Kevin Ryan Parks, Yen-Hong Kuo, John Mihran Davis, Brittany O’ Brien, Ellen J Hagopian

**Affiliations:** 1 Department of Surgery, Jersey Shore University Medical Center; 2 Office of Academic Affairs, Jersey Shore University Medical CenterNeptune; 3 Department of Surgery, Robert Wood Johnson Medical SchoolNew Brunswick, NJ, USA

## Abstract

**Background:** Laparoscopic liver resection is growing in popularity, but the long-term outcome of patients undergoing laparoscopic liver resection for malignancy has not been established. This paper is a meta-analysis and compares the long-term survival of patients undergoing laparoscopic (LHep) versus open (OHep) liver resection for the treatment of malignant liver tumours.

**Methods:** A PubMed database search identified comparative human studies analysing LHep versus OHep for malignant tumours. Clinical and survival parameters were extracted. The search was last conducted on 18 March 2012.

**Results:** In total, 1002 patients in 15 studies were included (446 LHep and 556 OHep). A meta-analysis of overall survival showed no difference [1-year: odds ratio (OR) 0.71, 95% confidence interval (CI) 0.42 to 1.20, *P* = 0.202; 3-years: OR 0.76, 95% CI 0.56 to 1.03, *P* = 0.076; 5-years: OR 0.8, 95% CI 0.59 to 1.10, *P* = 0.173]. Subset analyses of hepatocellular carcinoma (HCC) and colorectal metastases (CRM) were performed. There was no difference in the 1-, 3-, and 5-year survival for HCC or in the 1-year survival for CRM, however, a survival advantage was found for CRM at 3 years (LHep 80% versus OHep 67.4%, *P* = 0.036).

**Conclusions:** Laparoscopic surgery should be considered an acceptable alternative for the treatment of malignant liver tumours.

## Introduction

For many different types of surgery, laparoscopic surgery has become widely accepted as a feasible alternative to traditional open procedures. However, a laparoscopic hepatectomy remains one of the last frontiers to be fully implemented. The first laparoscopic hepatectomy, performed in 1992 by Gagner *et al*., consisted of a non-anatomic wedge resection for a benign tumour.^1^ The procedure was originally thought to be suited only for benign tumours, wedge resections or left lateral sectionnectomies. This approach has since evolved to encompass more difficult anatomic resections, including formal left and right hepatectomies, as well as extended hepatectomies. Several real or perceived factors have hindered the popularity of laparoscopic liver procedures, including the technical demand associated with a parenchymal resection, concerns regarding tumour-free margins, the possibility of uncontrolled bleeding and the opportunity for port-site metastases. In spite of these challenges, at the beginning of the new millennium, several centres have reported their experiences in laparoscopic liver resections. After initial reports, an increasing number of studies indicated that the laparoscopic approach was feasible. In an effort to better define the current position and future of laparoscopic liver resections, a consensus conference was convened in Louisville, Kentucky in 2008. Expert panels during this consensus concluded that the laparoscopic approach is safe and effective when performed by trained and experienced surgeons. The panels found that a laparoscopic hepatectomy offers many of the same benefits as other forms of minimally invasive surgery, however, long-term oncological outcomes were restricted to single-centre studies with limited follow-up.^2^

In 2009, 2804 laparoscopic liver cases were reported in a systematic review, which showed that peri-operative factors either showed no difference or favoured laparoscopy. The review also noted the oncological outcome of the laparoscopic was comparable to the open approach, however, only one study reported 5-year survival.^3^ A more recent meta-analysis by Zhou *et al*. found that a laparoscopic hepatectomy is not only comparable, but possibly superior to the traditional open procedure in the peri-and post-operative outcomes in hepatocellular carcinoma (HCC), where they report lower blood loss with no significant difference in terms of oncological resection margins. In this study, Zhou included 5-year survival data from four studies.^4^ A meta-analysis by Croome and colleagues evaluating benign and malignant tumorectomies confirmed the peri-operative outcomes of previous meta-analyses. Additionally, Croome included long-term data in a 2–5 year range from six studies, where not all the studies included in this calculation reported 5-year data.^5^

The primary outcomes of the previous meta-analyses were peri-operative or immediate post-operative outcomes. What has yet to be confirmed is the long-term outcome of patients undergoing a laparoscopic (LHep) versus an open hepatectomy (OHep) for malignant solid liver tumours. Previous meta-analyses have touched on long-term outcome, but went unstressed by the authors or may have lacked the number of studies to make meaningful conclusions. This study will analyse the available current data to determine the two procedures’ long-term outcomes. The aim of this study is to perform a comprehensive meta-analysis of overall long-term survival of those undergoing a laparoscopic as compared with an OHep for malignant tumours.

## Methods

### Literature search

A PubMed database search was performed to identify comparative studies analysing laparoscopic versus open hepatectomies for malignant tumours. Medical Subject Heading keywords, ‘laparoscopy’ and ‘hepatectomy’ were used in combination with publication type ‘comparative study’ to search the database. A more extensive text word search in MEDLINE was conducted using combinations of the keywords, ‘laparoscopic,’ ‘laparoscopy,’ ‘minimally invasive,’ ‘hepatectomy,’ ‘hepatic lobectomy,’ ‘liver lobectomy,’ ‘hepatic segmentectomy,’ ‘liver segmentectomy,’ ‘liver sectionectomy,’ ‘hepatic sectionectomy,’ ‘hepatic resection,’ ‘liver resection,’ ‘liver surgery’ and ‘hepatic surgery.’ The search was restricted to only human studies. The most recent search was conducted on 18 March 2012.

### Data extraction

The meta-analysis was performed in line with previously established guidelines.^4^ Data were independently extracted from each study by two reviewers (K.R.P. and E.J.H.) for the parameters: first author’s name, date of publication, study design, gender, age, number of patients for each procedure, matching method, pathology, operative parameters, oncological clearance, conversion rates, length of hospital stay, post-operative mortality and long-term survival (1, 3 or 5 years). Studies were evaluated for reporting data as either standard deviation or standard error of the mean. When the use of either was not specified or could not be verified, the reported measurements were assumed as standard deviation for our analysis. The reviewers were in 100% agreement upon completion of extraction.

### Inclusion criteria

Studies were included in the analysis if: (i) they were comparative human studies focusing on LHep versus OHep for solid tumours; and (ii) the 1-, 3-, or 5-year survival of patients with malignancies could be extracted or calculated.

### Exclusion criteria

Criteria for exclusion from the analysis included studies that: (i) did not compare LHep and OHep, or where the operative approach was not clearly documented; (ii) there was an inability to extract essential data or conduct a statistical analysis; (iii) contained insufficient data on overall survival; or (iv) included repeat hepatectomies or hepatectomies performed prior to liver transplantations.

### Quality assessment

All studies included were scored based on the Newcastle–Ottawa Scale (NOS) for assessing the quality of the studies. The NOS implements a ‘star system’, grading each study in three distinct areas: selection of the groups, comparability of those groups and the degree or quality to which the exposure or outcome of interest was expressed. A maximum total of nine stars can be allocated to one study, with ‘Selection’ a possible four, ‘Comparability’ a possible two and ‘Outcome’ a possible three. Tumour size was chosen as the most significant factor to be controlled in order to merit one star in ‘Comparability’.^6^

### Outcomes

The outcomes of interest encompassed operative parameters, post-operative parameters and long-term survivals. Operative parameters including operative time, blood loss and oncological margins were tabulated. Post-operative parameters included length of hospital stay and post-operative mortality. The analyses of overall survival for all malignancies were performed at 1-, 3-and 5-years. Two subgroup analyses for HCC and colorectal metastases (CRM) were also available for calculation of the overall 1-, 3-or 5-year survivals.

### Statistical analysis

The means and standard deviations of each patient’s age, blood loss, operative time and the frequency of gender were extracted from each study; the DerSimonian–Laird estimate of the mean age difference between groups was then calculated.^7^ The pooled proportion of females in each group was calculated using the Freeman–Tukey Double arcsine transformation.^8^ If no survival rates were reported, the proportion of surviving patients at each point was estimated based on the Kaplan–Meier curve. The estimated number of surviving patients at the time of interest and the total numbers of patients in each group were used to estimate the pooled survival rates. The funnel plot and Egger’s regression test were used to assess the publication bias.^9^,^10^ The between-study heterogeneity was quantified using the *I*^2^ (Higgins *et al*.^11^) statistic and the test of heterogeneity was performed using Q statistic.^12^

Pooled odds ratios (ORs) with 95% confidence intervals (CIs) were calculated using the DerSimonian–Laird random-effect model.^7^ The results are presented in the forest plots using a diamond shape in which the width represents the 95% CI. Each square in the chart area represents the OR of a study at its centre, with the area proportional to its weight, which is the inverse of the study variance. Statistical analysis was performed using meta package in R language.^13^,^14^ All of the tests were two-sided. A *P*-value of 0.05 or less was considered an indication of statistical significance.

## Results

### Eligible studies

**Figure 1:**
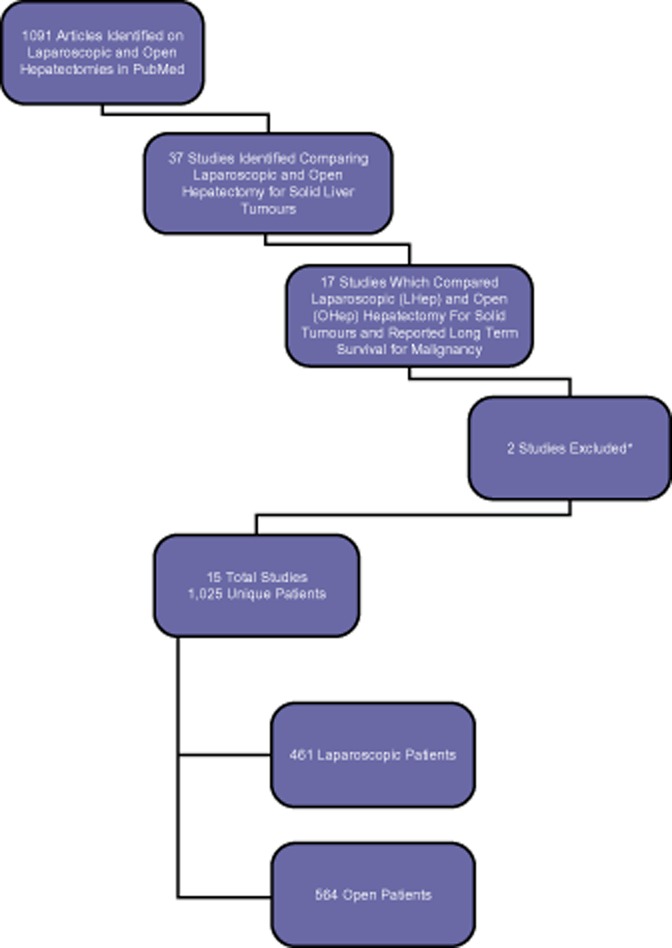
Selection of included studies for meta-analysis

The primary literature search garnered a total of 1091 abstracts. After review, abstracts were then narrowed down to 37 articles that compared LHep and OHep for solid liver tumours. Twenty studies were excluded from the analysis owing to the omission of survival data. Of the remaining 17 articles, 2 articles^15^,^16^ were found to, after contact with the respective authors, contain data from patients that were reported again in more recent studies.^17^,^18^ These more recent studies contained additional data on long-term results and thus are included in this meta-analysis. In total, 15 articles were identified to meet inclusion criteria for this meta-analysis^17^–^31^ (see Fig. 1).

The articles reported a total 1002 patients: 446 of whom underwent LHep whereas 556 underwent OHep. Of those, 308 patients underwent LHep whereas 404 patients underwent OHep for HCC, while 94 patients versus 100 underwent LHep versus OHep for CRM, respectively. One study presented did not specify tumour type of 47 malignant open patients.^24^ In addition, seven other types of malignancies were reported within the laparoscopic group, including five intrahepatic cholangiocarcinomas,^21^,^24^ five breast cancer,^21^,^24^ three melanoma,^24^ one primary sarcoma,^24^ one ovarian metastasis,^24^ one neuroendocrine metastasis^24^ and one sarcoma.^24^ Several of the studies focused on a single type of cancer, of which 10 studies reported only HCC, and three studies reported only CRM.

### Pre-operative parameters

In 11 studies, the two groups were matched for specific characteristics (see Table 1), whereas in the remaining four studies of a retrospective non-matched design, the study groups were similar in distribution of age, gender and tumour size. The distribution of age analysed in eight studies^20^,^22^,^23^,^25^,^28^–^31^ could be included within a statistical analysis of the age of patients undergoing a hepatectomy, which was not significant (*P* = 0.217). The other seven studies could not be included within the statistical analysis because of lack of detail. However, each of the studies individually determined that age and gender was not significantly different between LHep and OHep.

**Table 1:** Individual study demographics, matching method and quality assessment

Reference Number	Author, publication year	Study design	Number of patients		Number of females		Age; mean ± standard deviation, (median), [range]		
			LHep	OHep	LHep	OHep	LHep	OHep	P-Value
17	Lee *et al*, 2011	Retrospective Matched	33	50	9	10	(59) [36–85]	(58.5) [32–81]	0.357
18	Abu Hilal *et al*, 2011	Retrospective Matched	21	26	11	13	(64) [26–82]	(63) [25–84]	0.431
19	Alemi *et al*, 2010	Retrospective	28	25	1	1	61.4 [37–81]	65.1 [49–88]	0.19
20	Belli *et al*, 2007	Retrospective Matched	23	23	10	9	59.5 ± 6.8 [49–72]	62.4 ± 7.7 [51–74]	0.381
21	Cai *et al*, 2008	Retrospective Matched	31	31	7	5	54.2 [23–81]	51.7 [38–71]	0.4
22	Castaing *et al*, 2009	Retrospective Matched	60	60	23	23	62 ± 11	62 ± 11	0.97
23	Hu *et al*, 2009	Retrospective Matched	30	30	10	11	46 ± 12**	48 ± 15**	n.s.
24	Ito *et al*, 2009	Retrospective Matched	37	47	*	*	*	*	*
25	Kaneko *et al*, 2004	Retrospective	30	28	12	10	59 ± 8**	61 ± 10**	n.s.
26	Lai *et al*, 2009	Retrospective Matched	25	33	7	12	(59) [35–79]	(59) [38–77]	0.3
27	Mala *et al*, 2002	Retrospective	13	14	4	4	(68) [55–73]	(59) [24–74]	NR
28	Sarpel *et al*, 2009	Retrospective Matched	20	56	5	11	63.8 ± 10.3	58.3 ± 11.0	0.054
29	Shimada *et al*, 2001	Retrospective	17	38	2	14	62 ± 9	63 ± 79	0.67
30	Tranchart *et al*, 2009	Retrospective Matched	42	42	15	14	63.7 ± 13.1	65.7 ± 7.1	0.96
31	Truant *et al*, 2011	Retrospective Matched	36	53	5	6	60.0 ± 10.2	63.3 ± 7.6	0.2

Reference Number	Pathology	Tumour size cm; mean ± standard deviation, (median), [range]			Matching***	Quality Assessment (#stars allocated)		
		LHep	OHep	P-Value		Selection	Comparability	Outcome
17	HCC Only	(2.5) [1.5–9]	(2.9) [1.2–9]	0.307	c,d,f,g	4	2	3
18	CRM Only	NR	NR	NR	a,b,j,r	4	1	2
19	HCC Only	4	5.2	0.12	q	4	1	2
20	HCC Only	3.1 ± 0.7 [1–3.9]	3.2 ± 0.7 [1.6-4.2]	0.983	a,b,c,d,e,f,o	4	2	3
21	Mixed Malignancies	NR	NR	NR	a,b,c,d,e	4	2	3
22	CRM Only	3.0 ± 1.6 (3.0) [0.5–8]	4.1 ± 3.1 (4.0) [0.8–16]	0.02	a,b,c,d,e,j,l,m,n	3	2	3
23	HCC Only	6.7 ± 3.1**	8.7 ± 2.3**	>0.05	c,d,j	4	2	3
24	Benign/ Mixed Malignancies	(3.3) [0.4–14.4]	(3.4) [0.9–13]	0.215	a,b,c,d,f,g,h,j,k,l	3	2	2
25	HCC Only	3.0 ± 0.8 **	3.1 ± 0.9 **	n.s.	q	3	2	2
26	HCC Only	(2.5) [1–7]	(2.6) [1–8]	0.54	c,e,f,p	4	2	2
27	CRM Only	(8) [3.5–16]	(9) [3–17]	n.s.	q	4	1	3
28	HCC Only	4.3 ± 2.1	4.3 ± 2.2	0.876	c,e	3	2	3
29	HCC Only	2.6 ± 0.9	2.5 ± 1.0	0.89	q	4	2	3
30	HCC Only	3.6 ± 1.8	3.7 ± 2.1	0.95	a,b,c,e,f,o	4	2	2
31	HCC Only	2.9 ± 1.2	3.1 ± 1.2	0.5	c,f,j	4	2	3

* Data could not be extracted for malignancies due to their combination with benign patients

** Data unclearly reported on the use of standard deviation versus standard error of the mean.

*** Matching method: a) age, b) gender, c) size of the tumour, d) location of the tumour, e) presence/severity of cirrhosis, f) type or resection, g) comorbidities, h) body mass index, i) pathology, j) number of tumours, k) histopathology of the background liver, l) distribution of metastases, m) initial resectability, n) prehepatectomy chemotherapy administration, o) ASA class, p) demographic data, q) patients not matched but were considered similar and r) operation type (right hepatic lobectomy)

A statistical analysis of gender in 14 studies was conducted, which overall was not significantly different (*P* = 0.951). The proportion of females undergoing LHep was 28.0% whereas OHep was 27.6%. All studies that recorded pre-operative tumour size reported that tumour size was not significantly different amongst the groups. Only eight studies could be included for the statistical analysis, which was not significantly different overall (LHep versus OHep weighted difference – 0.23 cm, 95% CI –0.52 to 0.06, *P* = 0.121).

Some of the studies in this meta-analysis presented unique concerns or findings with regard to tumour size. Castaing *et al*. recorded tumour sizes for patients before surgery to be used for matching purposes, yet found after surgery that actual tumour sizes based on pathology were significantly larger in the open cohort (*P* = 0.02) As tumour size was matched before treatment, no selection bias had taken place.^22^ Hu *et al*. reported that tumour size was not significantly different. However, our own analysis shows a difference in Hu’s study between the two groups, with a mean difference of −2.00 cm (LHep versus OHep 95% CI −3.38 to −0.62). Another study, Ito *et al*.,^24^ was not included in the analyses of age, gender, and tumour size, owing to an inability to extract these data for patients undergoing a resection for malignancy only. This study, however, determined their LHep and OHep groups to be similar. Age, gender and tumour sizes were not significantly different, and tumor sizes were matched for Hu’s and Ito’s studies. Ito’s study combined benign and malignant tumours to determine short-term outcome. However, this study reported long-term survival analyses of patients with malignancy only. Therefore, Ito’s study was not included in the peri-or post-operative measures, but was used for long-term survival tabulations in this study.

### Operative parameters

Blood loss was extracted from five studies for statistical analysis.^20^,^23^,^25^,^30^,^31^ Blood loss was significantly lower in the group undergoing LHep than in the group undergoing OHep as reported by the mean difference in blood loss (in ml) in the two procedures (LHep versus OHep weighted mean difference 103.71 ml, 95% CI 0.73 to 206.70 ml, *P* = 0.048). Operative times were extracted from six studies and were included in the analysis.^20^,^22^,^23^,^28^,^30^,^31^ There was no significant difference in the operative time between the two groups (LHep versus OHep weighted mean difference 5.82 h, 95% CI −7.59 to 19.23, *P* = 0.395).

Differences in the operative margins could not be analysed because of differences in the definitions of positive surgical margins and the variability of reporting. The most common definition of a positive margin was less than 1 mm of tumour-free parenchyma between the lesion and resection line. However, several previous studies included within this meta-analysis defined positive margins as greater than 1 mm. Sarpel *et al*. chose to define positive margins as any margin less than 3 mm and reported a 10% positive margin for LHep and 26.8% for OHep.^28^ Similarly, Shimada *et al*. reported a 41.2% positive margin for LHep and a 50% positive margin for OHep using a definition of less than 5 mm.^29^ Although we could not evaluate these data in our meta-analysis, there was no significant difference in the oncological clearance in each individual study.

Conversion to a laparotomy was necessary in 46 cases (4.2%) in 11 studies.^17^,^18^,^20^–^22^,^25^,^26^,^28^,^30^,^31^ Conversions were caused by 17 cases of intra-operative bleeding,^17^,^21^,^22^,^24^,^26^,^30^,^31^ 7 cases due to the extent of the tumour,^18^,^22^,^24^ 5 adhesions,^22^,^24^ 3 cases due to technical difficulties,^31^ 2 cases of failed hilar dissection,^18^ 2 cases of indeterminate oncological clearance,^24^ 1 case due to peritoneal tumour implants,^22^ 1 case for the control of the right hepatic vein,^22^ 1 case due to failure to locate the tumour,^18^ 1 case of vascular proximity^24^ and 1 case insufficient exposure.^20^ The cause for conversion was not reported in 5 cases.^25^,^28^

### Post-operative parameters

Ten of the 15 studies demonstrated a significant difference in the length of hospital stay for patients, favouring LHep.^17^,^18^,^21^,^23^–^25^,^27^,^29^–^31^ The remaining five studies either reported no significant difference^19^,^20^,^22^,^26^or did not report a *P*-value.^28^

The overall 30-day mortality was 1.4% (15 deaths), which included 3 (0.7%) deaths in the LHep group, and 9 (1.6%) deaths in the OHep group. Causes of death in the LHep group included clip failure from a portal vein branch,^22^ liver failure^30^ and acute respiratory distress syndrome.^20^ Noted causes of death for OHep included multi-organ dysfunction,^30^ pseudo-membranous colitis^22^ and four cases of liver failure.^31^ The causes for the remaining deaths in patients undergoing OHep were not reported.^26^ One study did not report 30-day mortality.^28^

### Survival parameters

#### All malignancies

**Figure 2:**
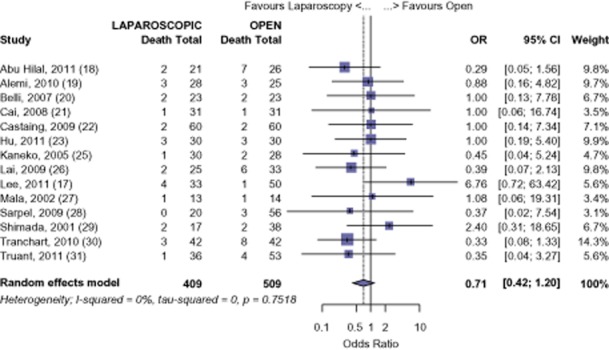
All-cancers: risk of death, 1-year

**Figure 3:**
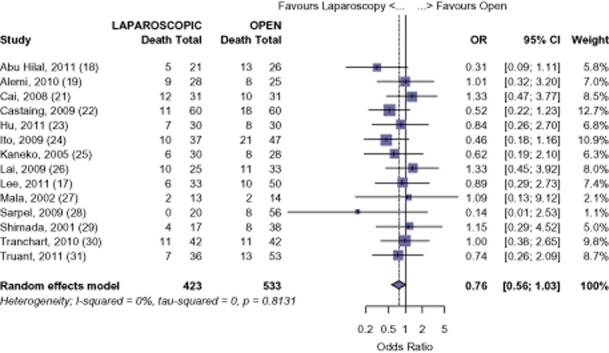
All-cancers: risk of Death, 3-year

**Figure 4:**
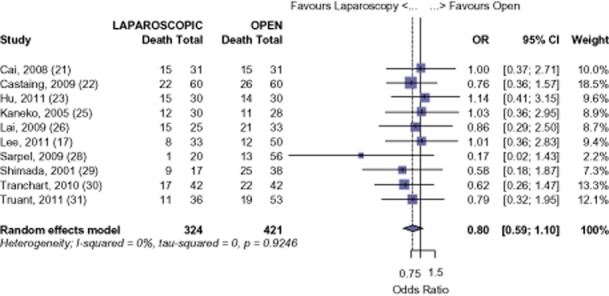
All-cancers: risk of death, 5-year

All 15 studies were analysed for long-term survival for all malignancies. Using available data from 14 studies, LHep was shown to have a 92.6% survival at 1 year, whereas OHep was comparable at 90.51% (OR 0.71, 95% CI 0.42 to 1.20, *P* = 0.202) (Fig. 2). Fourteen studies had available data for 3-year survival with a 76.5% survival for LHep and 71.6% survival for OHep (OR 0.76, 95% CI 0.56 to 1.03, *P* = 0.076) (Fig. 3). Five-year survival was reported in 10 studies. At 5 years, the meta-analysis found a 61.7% survival for LHep and 56.4% for OHep (OR 0.80, 95% CI 0.59 to 1.10, *P* = 0.173) (Fig. 4). No significant difference was found in the 1-, 3-or 5-year survival for all malignant tumours after LHep versus OHep. There was no evidence of publication bias amongst the studies based on the funnel plot and the weighted linear regression approach [1-year: *t* = 1.170, degrees of freedom (d.f.) = 12, *P* = 0.265; 3-years *t* = −0.405, d.f. = 12, *P* = 0.693; 5-years *t* = −1.49, d.f. = 8, *P* = 0.175; funnel plots not shown).

Because of the various tumour types reported in the studies, subset analyses of the two most commonly encountered tumours, HCC and CRM, were performed.

#### HCC

**Figure 5:**
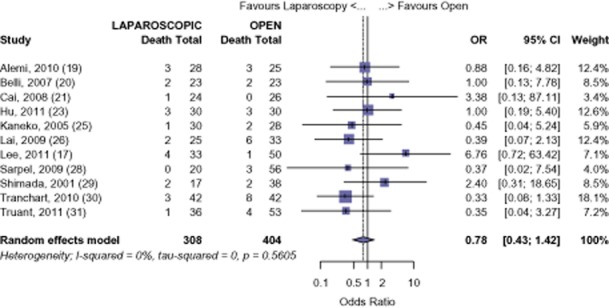
Hepatocellular carcinoma (HCC): risk of death, 1-year

**Figure 6:**
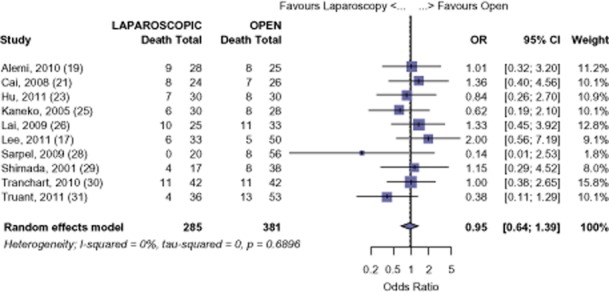
Hepatocellular carcinoma (HCC): risk of death, 3-year

**Figure 7:**
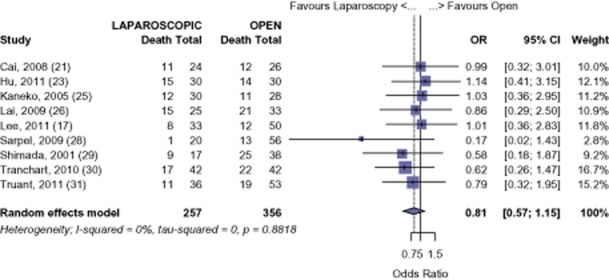
Hepatocellular carcinoma (HCC): risk of death, 5-year

The survival of those undergoing LHep for HCC was 92.0%, 77.7% and 61.9% at 1-, 3-and 5-years. The survival of those undergoing OHep for HCC was similar: 91.3%, 76.5% and 56.5% at 1-, 3-and 5-years, respectively (1-year: OR 0.78, 95% CI 0.43 to 1.42, *P* = 0.422; 3-years: OR 0.95, 95% CI 0.64 to 1.39, *P* = 0.778; 5-years: OR 0.81, 95% CI 0.57 to 1.15, *P* = 0.239) (Figs 5–7). There was no evidence of publication bias amongst the studies based on the funnel plot and the weighted linear regression approach (1-year: *t* = 1.206, d.f. = 9, *P* = 0.259; 3-years: *t* = −1.465, d.f. = 8, *P* = 0.181; 5-years: *t* = −1.855, d.f. = 7, *P* = 0.106; funnel plots not shown).

#### Colorectal liver metastases

**Figure 8:**
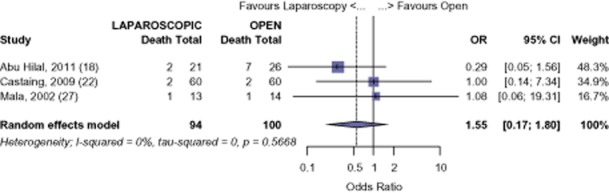
Colorectal metastases (CRM): risk of death, 1-year

**Figure 9:**
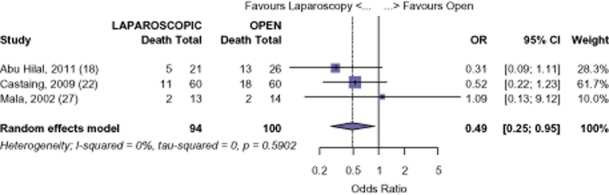
Colorectal metastases (CRM): risk of death, 3-year

Although long-term data are limited, there is comparable survival at 1-year after LHep versus OHep for CRM (OR 0.55, 95% CI 0.17 to 1.80, *P* = 0.325). (Fig. 8) However, the analysis shows a significant difference in survival at 3 years (OR 0.49, 95% CI 0.25 to 0.95, *P* = 0.036). (Fig. 9) The survival of those undergoing LHep for CRM was 93.8% and 80.0% at 1 and 3 years, respectively. The survival of those undergoing OHep was 88.1% and 67.4% at 1-and 3-years, respectively.^18^,^22^,^27^ There was no evidence of publication bias amongst the studies based on the funnel plot and the weighted linear regression approach (1-year: *t* = 1.067, d.f. = 1, *P* = 0.480; 3-years: t = 0.405, d.f. = 1, *P* = 0.755; funnel plots not shown). Survival figures for CRM at 5 years were not analysed as only one study reported 5-year survival.^22^

## Discussion

While the efficacy of a laparoscopic hepatectomy has been questioned in the past, this study demonstrates that LHep is comparable to OHep in treating malignancy. The success of LHep and the reservation with which the procedure has been viewed have spurred a collective need to examine the true benefits of a minimally invasive procedure. This meta-analysis sought to demonstrate that LHep is an alternative to OHep, a question that was previously explored regarding a laparoscopic resection for colon cancer. Similar to LHep, laparoscopic colectomy was under great scrutiny, with regard to long-term survival of patients, owing to a concern for local and trocar-site recurrences. In a breakthrough study, Fleshman *et al*. established that a laparoscopic procedure does not ‘adversely affect the oncologic outcome of the patient’.^32^ The study showed no difference in the overall 5-year survival rates for laparoscopic as opposed to open colectomy. One criticism this study has received was the inability to attain its goal of garnering 1200 patients. Several reasons contributed to this shortcoming, the most notable of which also applies to the present study: patients who are suitable candidates for laparoscopic procedures are less likely to willingly undergo traditional open procedures and forgo the well-known short-term benefits of laparoscopy. It is for this reason that a prospective randomized clinical trial may never come to fruition in order to compare the long-term survival rates of laparoscopic and open procedures.

Similarly, in 2006, a meta-analysis from Simillis *et al*. took a step towards validation of LHep as an alternative to OHep, but only looked at peri-operative outcomes without long-term survival.^33^ In agreement with the findings of Simillis *et al*., this study found a lower blood loss for those undergoing LHep and no difference in operative time between the two groups. Owing to the difference in data reporting, not all data could be entered into a comprehensive statistical analysis. However, the objective of our meta-analysis to report on long-term survival was achieved.

The aim in this meta-analysis was to investigate the use of the laparoscopic approach to liver resection for malignancy, focusing on long-term survival. The articles included within this analysis contained 10 studies reporting on only HCC and three studies that reported only CRM. The two other studies reported survival based on multiple types of hepatic malignancies.^21^,^24^ This meta-analysis demonstrates that LHep is comparable to OHep at 1-, 3-and 5-years and provides evidence that LHep is comparable to OHep with regard to long-term results. However, although overall survival is not different, it is possible that a selection bias may have occurred in the studies that did not utilize the matching criteria. However, each of these studies compared their study groups to ensure comparability between the groups.

Within this study, we included a subset analysis of HCC and CRM. Of the patients undergoing a resection for HCC, 308 patients underwent LHep, whereas 404 patients underwent OHep. The survival of patients undergoing LHep for HCC was comparable to those undergoing OHep at 1, 3 and 5 years. Given these data and the benefit of the laparoscopic approach in cholecystectomy patients with cirrhosis,^32^–^35^ LHep should be considered as the leading approach for those patients with cirrhosis. LHep is at least comparable in terms of survival to OHep at 1-year and favourable at 3-years for CRM. A decrease in stress as compared with a laparotomy,^36^ which alters the immunosuppressive effects of surgery,^37^ may explain the possible advantage of the laparoscopic as opposed to the open approach. Because only three studies were available for meta-analysis, insufficient data exists at this time to clearly favour LHep for CRM. Further research needs to be conducted before recommendations are made for CRM.

The gender distribution in the studies revealed a larger proportion of male patients, which is similar to that of the nation-wide patient population of nearly 3:1 male/female.^38^ Two studies had particularly low numbers of females,^19^,^29^ with one of these conducted at a Veterans Affairs hospital, which reported a ‘preponderance of elderly males’.^19^

Several limitations of this study must be taken into account. The peri-and post-operative data were reported in several different ways, and therefore, could not all be included in the statistical analyses. A statistical analysis was not done for the oncological margin, owing to differences both in reporting and defining a positive margin.

This meta-analysis has shown that LHep is comparable to OHep for all malignancies at 1-, 3-and 5-year overall survival, as well as for HCC. Although further study should be undertaken in those with CRM, the currently available data demonstrates that survival is comparable after LHep at 1 year and favourable at 3 years. Thus, LHep should be considered as an acceptable alternative for the treatment of malignant liver tumours.

## Conflicts of interest

None declared.
